# Volume loss in the left anterior‐superior subunit of the hypothalamus in amyotrophic lateral sclerosis

**DOI:** 10.1111/cns.14801

**Published:** 2024-06-17

**Authors:** Sadegh Ghaderi, Farzad Fatehi, Sanjay Kalra, Sana Mohammadi, Fariba Zemorshidi, Mahtab Ramezani, Omid Hesami, Saharnaz Pezeshgi, Seyed Amir Hossein Batouli

**Affiliations:** ^1^ Department of Neuroscience and Addiction Studies, School of Advanced Technologies in Medicine Tehran University of Medical Sciences Tehran Iran; ^2^ Neuromuscular Research Center, Department of Neurology, Shariati Hospital Tehran University of Medical Sciences Tehran Iran; ^3^ Neurology Department University Hospitals of Leicester NHS Trust Leicester UK; ^4^ Neuroscience and Mental Health Institute University of Alberta Edmonton Alberta Canada; ^5^ Division of Neurology, Department of Medicine University of Alberta Edmonton Alberta Canada; ^6^ Department of Neurology Mashhad University of Medical Sciences Mashhad Iran; ^7^ Department of Neurology Shahid Beheshti University of Medical Sciences Tehran Iran

**Keywords:** ALS, atrophy, hypothalamus, MRI

## Abstract

**Background and Objective:**

Amyotrophic lateral sclerosis (ALS) causes motor neuron loss and progressive paralysis. While traditionally viewed as motor neuron disease (MND), ALS also affects non‐motor regions, such as the hypothalamus. This study aimed to quantify the hypothalamic subregion volumes in patients with ALS versus healthy controls (HCs) and examine their associations with demographic and clinical features.

**Methods:**

Forty‐eight participants (24 ALS patients and 24 HCs) underwent structural MRI. A deep convolutional neural network was used for the automated segmentation of the hypothalamic subunits, including the anterior‐superior (a‐sHyp), anterior‐inferior (a‐iHyp), superior tuberal (supTub), inferior tuberal (infTub), and posterior (posHyp). The neural network was validated using FreeSurfer v7.4.1, with individual head size variations normalized using total intracranial volume (TIV) normalization. Statistical analyses were performed for comparisons using independent sample *t*‐tests. Correlations were calculated using Pearson's and Spearman's tests (p < 0.05). The standard mean difference (SMD) was used to compare the mean differences between parametric variables.

**Results:**

The volume of the left a‐sHyp hypothalamic subunit was significantly lower in ALS patients than in HCs (*p* = 0.023, SMD = ‐0.681). No significant correlation was found between the volume of the hypothalamic subunits, body mass index (BMI), and ALSFRS‐R in patients with ALS. However, right a‐sHyp (*r* = 0.420, *p* = 0.041) was correlated with disease duration, whereas right supTub (*r* = −0.471, *p* = 0.020) and left postHyp (*r* = −0.406, *p* = 0.049) were negatively correlated with age. There was no significant difference in the volume of hypothalamic subunits between males and females, and no significant difference was found between patients with revised ALS Functional Rating Scale (ALSFRS‐R) scores ≤41 and >41 and those with a disease duration of 9 months or less.

**Discussion and Conclusion:**

The main finding suggests atrophy of the left a‐sHyp hypothalamic subunit in patients with ALS, which is supported by previous research as an extra‐motor neuroimaging finding for ALS.

## Graphical abstract

This study reveals significant atrophy in the left anterior‐superior hypothalamus of ALS patients compared with healthy controls, highlighting the disease's broader neurological impact beyond motor neuron degeneration.

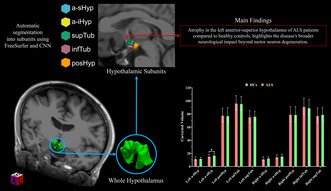

## 1 INTRODUCTION

Neurodegeneration typically affects various cortical and subcortical areas, including the hypothalamus.^1^ Amyotrophic lateral sclerosis (ALS) is the third most common neurodegenerative disease globally, after Alzheimer's disease (AD) and Parkinson's disease (PD).^2^, ^3^ It is characterized by progressive loss of motor neurons, causing focal muscle weakness, rapid muscle wasting, and paralysis.^4^ More than 60% of patients die within 3–5 years of diagnosis.^5^ Despite efforts to understand the complexity of this disease, mechanisms underlying progressive neuronal death remain unclear.

The hypothalamus is a crucial component of the neuroendocrine system in the brain and is gaining recognition for its significance in neurodegenerative diseases, specifically ALS,^6^, ^7^, ^8^ and other diseases such as Huntington's disease (HD)^9^ and behavioral‐variant frontotemporal dementia (bvFTD).^10^ It functions as a visceral and autonomic regulatory center, controlling essential activities, such as energy balance, thermoregulation, and emotional responses through its diverse neuronal subpopulations.^11^, ^12^, ^13^

Inflammation is a significant factor in the development of neurodegenerative diseases, such as AD, PD, ALS, and Huntington's disease (HD).^14^ The integrity of the hypothalamus is critical, as even the pre‐symptomatic stages of genetic ALS variants with hexanucleotide expansion of chromosome 9 open reading frame 72 (C9orf72) show hypothalamic atrophy, which is believed to contribute to the metabolic and autonomic disturbances of the disease.^15^ Metabolic dysfunction underlying ALS is still not understood at the neural mechanism level.^16^ However, emerging findings suggest that the hypothalamus, which is responsible for regulating sleep–wake cycles, is involved in the development of ALS‐associated sleep problems.^11^ Moreover, the hypothalamus acts as a hub for a vast network of neural circuits that regulate food‐related behavior.^17^ Recent studies have shown that inflammation is not only a consequence of neurodegeneration but also a key contributor to this process. In the case of ALS, various research teams have reported that patients with ALS have a significantly reduced hypothalamus volume compared with control subjects.^7^, ^15^, ^17^

The hypothalamic atrophy changes and connectivity with other non‐motor regions suggest a complex involvement in ALS pathophysiology.^6^, ^18^, ^19^ Moreover, connected to other subcortical nuclei and structures, such as limbic structures, the hypothalamus is a central hub that integrates aspects of physiological processes.^20^ Furthermore, disruptions in orbitofrontal‐hypothalamic projections have been identified, indicating structural changes that may contribute to the hypermetabolic phenotype observed in ALS.^21^ Thus, these findings highlight the need for further in‐depth exploration of subunit volumes and their role in the clinical presentation of ALS.

Despite previous efforts, current research on hypothalamic subunits in patients with ALS is notably limited and inconsistent, for example, regarding alterations in the volume of the hypothalamic subunits.^22^, ^23^, ^24^ Given the complexity of ALS and the multifaceted role of the hypothalamus in the regulation of essential physiological processes, it is imperative to explore the patterns of hypothalamic damage and its clinical implications. Thus, this study aimed to determine the volumes of hypothalamic subunits and their correlation with demographic and clinical parameters in patients with ALS compared with healthy controls (HCs).

## 2 METHODS

### 2.1 Ethics approval/statement

The study was approved by the Ethics Committee of the Tehran University of Medical Sciences (Ethical Code: IR.TUMS.MEDICINE.REC.1400.1173). Written consent was obtained from all participants with potentially identifiable images or data. Information was collected only after a briefing, and informed written consent was obtained from the patients and healthcare providers.

### 2.2 Participants

Forty‐eight participants, including 24 patients diagnosed with ALS and 24 HCs, were recruited from the ALS Clinic at Shariati Hospital, Tehran University of Medical Sciences, Tehran, Iran. The HCs were carefully matched with ALS patients regarding age, sex, and body mass index (BMI). The patients underwent thorough clinical examination to ensure the absence of neurological, psychiatric, or cognitive disturbances.

Standard diagnostic assessments included clinical interviews, medical and neurological examinations, and structural MRI. Participants with significant sensory complaints; other nervous system disorders; or a history of nerve, spinal cord, psychiatric disorders, or brain trauma were excluded from the study.

The diagnosis of the patients was established through a multidisciplinary consensus led by a senior neurologist, adhering to the prevailing clinical diagnostic criteria. The patients fulfilled the definite or probable ALS diagnostic criteria according to the Awaji criteria^25^ and maintained their routine medications throughout the study. Functional impairment in patients with ALS was quantified using the revised ALS Functional Rating Scale (ALSFRS‐R).

### 2.3 Imaging

#### 2.3.1 MRI acquisition

The MRI images were acquired using a Siemens scanner (Prisma, 2016) equipped with a 64‐channel, generalized autocalibrating partial parallel acquisition (GRAPPA) head coil for parallel imaging. High‐resolution structural MRI data were obtained using a 3D T1‐weighted magnetization‐prepared gradient echo imaging (MPRAGE) sequence consisting of 176 axial slices with a voxel resolution of 1 × 1 × 1 mm^3^ and no gap. The following imaging parameters were used: repetition time, 1840 ms; echo time = 2.43 ms, inversion time, 900 ms; slice thickness, 1 mm; flip angle, 8°; field of view (FOV) read, 255 × 255 mm^2^; and FOV phase, 100%.

#### 2.3.2 MRI data analysis and automated segmentation of the hypothalamus

T1‐weighted images were pre‐processed using the FreeSurfer v7.4.1 ‘recon‐all’ pipeline (https://surfer.nmr.mgh.harvard.edu/fswiki/recon‐all). The pre‐processed structural data were then segmented to extract the entire hypothalamus and its nuclei, including the entire left and right hypothalamus, as well as five subunits: anterior‐superior (a‐sHyp) [preoptic area and paraventricular nucleus (PVN)], anterior‐inferior (a‐iHyp) [suprachiasmatic nucleus (SCN) and supraoptic nucleus (SON)], superior tubular (supTub) [dorsomedial nucleus, PVN, and lateral hypothalamus], inferior tubular (infTub) [infundibular (or arcuate) nucleus, ventromedial nucleus, SON, lateral tubular nucleus, and tuberomamillary nucleus (TMN)], and posterior (posHyp) [mamillary body (including medial and lateral mamillary nuclei), lateral hypothalamus, TMN].^26^ We conducted thorough visual quality checks on all scans and segmentations to ensure a high quality. One ALS participant was excluded from the analyses because of motion artifacts.

This approach uses an automated tool based on a deep convolutional neural network (CNN) on volumetric MRI scans to parcellate the gray matter volumes of subregions of the hypothalamus. Although the segmented subregions overlap with the more detailed nuclei of the hypothalamus, they do not isolate the functional or anatomical regions.^26^

In volumetric brain imaging analysis, it is commonly assumed that the volume of the brain structures is directly related to the total intracranial volume (TIV) proportionally or linearly.^27^ Therefore, before conducting the statistical analysis, the volume of the hypothalamic subunits for each participant was corrected based on the estimated TIV using the “aseg” atlas and the following equation: (volume of each subunit/total intracranial volume (TIV)) × 10^6^. This adjustment allows for the correction of variations in individual head size and brain volume.

### 2.4 Statistical analyses

Variable normality was assessed using the Shapiro–Wilk test. To assess differences in hypothalamic subregion volumes among cases and controls, as well as between sexes, disease duration with a cutoff of nine (median disease duration), and ALSFRS‐R with a cutoff of 41 (median ALSFRS‐R), we employed an Independent Samples *T*‐test for parametric variables. The magnitude of the difference was determined using the standardized mean difference (SMD) effect size.^28^

In the univariate analysis, Pearson's correlations were applied to scrutinize the associations between hypothalamic subregion volumes and age, BMI, disease duration, and ALSFRS‐R under continuous, normally distributed, and linearly related data. Spearman's correlations were performed in instances where these criteria were not met. The significance level was set at *p* < 0.05. All statistical analyses were performed using SPSS V.27.0 (IBM SPSS Statistics) software, and figures were created using GraphPad Prism 10 (GraphPad Software, San Diego, CA, USA).

## 3 RESULTS

### 3.1 Demographics

**FIGURE 1:**
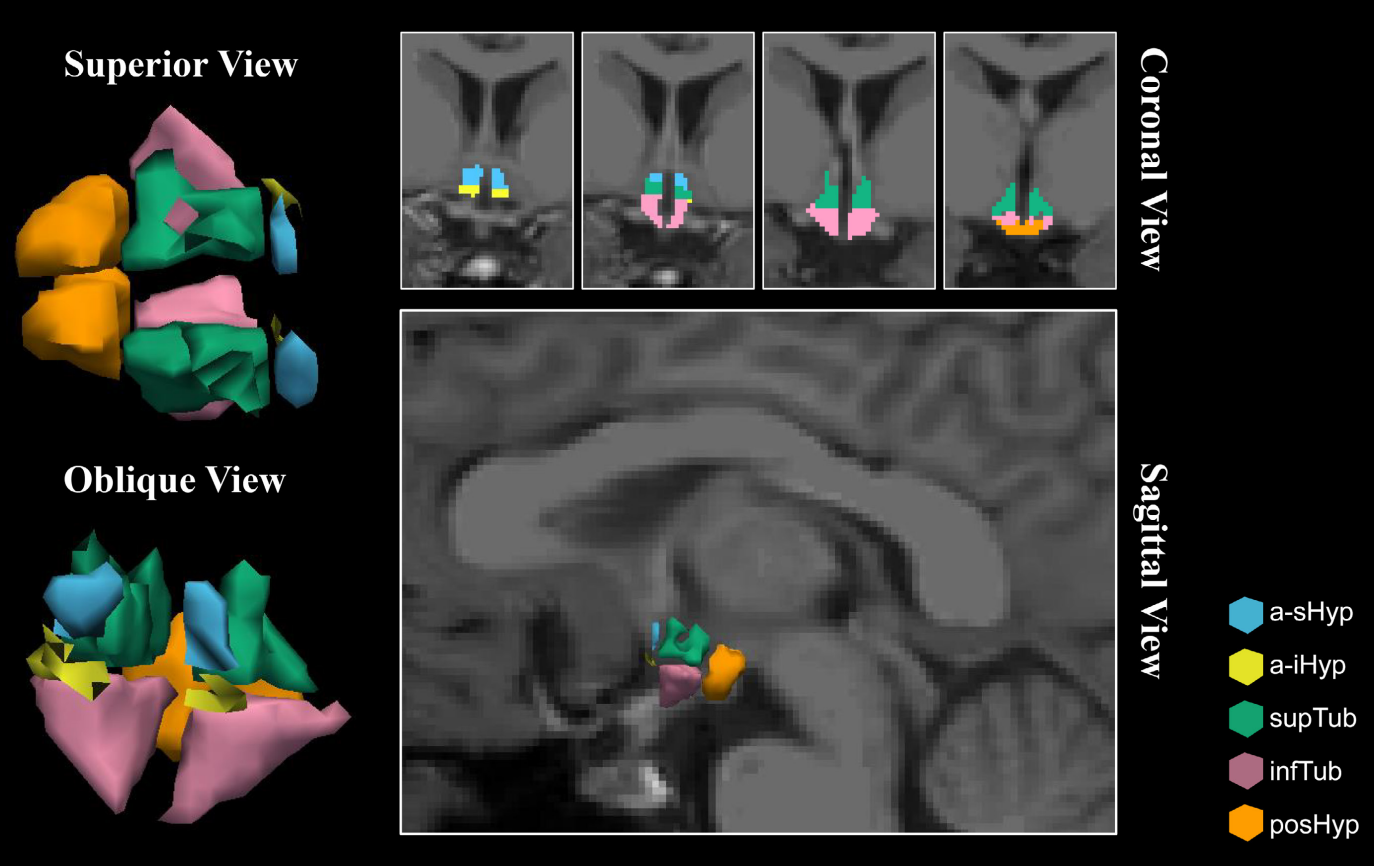
Automatic segmentation of the hypothalamic subunits for a single participant. The subunits included the anterior‐superior (a‐sHyp), anterior‐inferior (a‐iHyp), superior tuberal (supTub), inferior tuberal (infTub), and posterior (posHyp).

Figure 1 illustrates the automated segmentation of the hypothalamic subunits for each participant. A total of 24 right‐handed patients and 24 right‐handed HCs were enrolled in this study. No significant differences in age, sex, or BMI were found between ALS patients and HCs (Table 1).

**TABLE 1:** Characteristics of the participants (patients with ALS and healthy controls).

	ALS (*N* = 24)	HC (*N* = 24)	*p*‐value*
Sex/male	16	16	1.00
Age, years	49.58 (10.64)	49.50 (10.72)	0.98
BMI, kg/m^2^	26.82 (4.90)	26.67 (3.28)	0.90
Disease duration, months	12.96 (11.03)	N/A	N/A
ALSFRS‐R	40.63 (4.37)	N/A	N/A

*Note*: Data presented as mean (SD) for continuous variables or n counts (%) for categorical variables.

Abbreviations: ALS, amyotrophic lateral sclerosis; ALSFRS‐R, revised ALS functional rating scale; BMI, body mass index; HC, healthy control; N/A, not applicable.

* *p* < 0.05.

### 3.2 Hypothalamic volumes

After conducting a normality test on all continuous variables, we discovered that the left posHyp, left infTub, and right posHyp volume variables in the control group, as well as the disease duration, ALSFRS‐R, right infTub volume, and whole left volume variables did not follow a normal distribution. However, the other variables showed a normal distribution.

#### 3.2.1 Comparison of hypothalamic subunit volume between ALS patients and healthy controls

**FIGURE 2:**
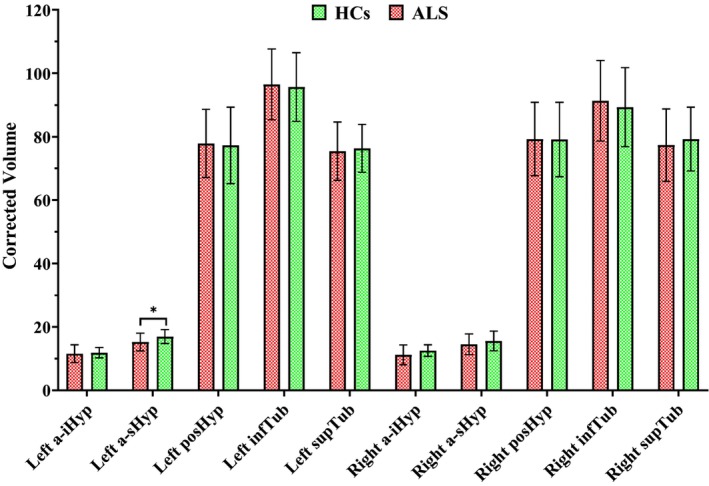
Hypothalamic subunit profiles of patients with amyotrophic lateral sclerosis (ALS) and healthy controls (HCs). Anterior‐superior (a‐sHyp), anterior‐inferior (a‐iHyp), superior tuberal (supTub), inferior tuberal (infTub), and posterior (posHyp). *p < 0.05. Corrected subunit volumes to total intracranial volume × 10^6^.

After comparing the volumes of all subunits of the hypothalamus between the patient and control groups, we found that only the volume of the left a‐sHyp was significantly lower in the patient group than in the control group (*p* = 0.023), with a standardized mean difference (SMD) of −0.681 (Table 2 and Figure 2).

**TABLE 2:** Volumetric comparisons of the hypothalamic subunits between the ALS patients and healthy controls.

Independent Samples *t*‐test^a^				
	ALS	HC	*p*‐value*	SMD^b^ (95% CI)
Left anterior‐inferior	11.58^c^ (2.81)^d^	11.88 (1.64)	0.656	−0.182
Left anterior‐superior	15.24 (2.80)	16.96 (2.20)	0.023	−0.681
Left posterior	77.89 (10.74)	77.25 (12.05)	0.846	0.056
Left tubular inferior	96.47 (11.17)	95.65 (10.85)	0.797	0.075
Left tubular superior	75.44 (9.21)	76.29 (7.53)	0.727	−0.101
Right anterior‐inferior	11.21 (3.12)	12.55 (1.80)	0.076	−0.746
Right anterior‐superior	14.51 (3.25)	15.57 (3.07)	0.251	−0.335
Right posterior	79.25 (11.59)	79.10 (11.73)	0.965	0.013
Right tubular inferior	91.32 (12.71)	89.31 (12.45)	0.583	0.160
Right tubular superior	77.33 (11.44)	79.21 (10.04)	0.549	−0.174
Whole left	276.63 (29.35)	278.03 (23.10)	0.855	−0.053
Whole right	273.63 (30.74)	275.74 (29.36)	0.809	−0.070
Whole	550.25 (57.06)	553.77 (49.49)	0.821	−0.066

Abbreviations: ALS, amyotrophic lateral sclerosis; HC, healthy control; SMD, standardized mean difference; TIV, total intracranial volume.

^a^ Two‐group comparisons were tested using the Independent Samples T‐test for parametric variables.

^b^ The effect size used was SMD (cut off; trivial: −0.19 to 0.19; small: 0.2 to 0.49 and −0.49 to −0.2; medium: 0.5 to 0.79 and −0.79 to −0.5; large: ≥0.8 and ≤−0.8).

^c^ Corrected subunit volumes to TIV × 10^6^.

^d^ Data presented as mean (SD).

* *p* < 0.05.

#### 3.2.2 Correlation between hypothalamic subunit volumes and demographic and clinical characteristics in ALS patients

No significant correlation was found between the volume of the hypothalamic subunits and BMI or ALSFRS‐R in patients with ALS. However, the right a‐sHyp was correlated with disease duration in patients with ALS (*r* = 0.420, *p* = 0.041). Furthermore, the right supTub (*r* = −0.471, *p* = 0.020) and left postHyp (*r* = −0.406, *p* = 0.049) were negatively correlated with the age of ALS patients.

#### 3.2.3 Comparison of hypothalamic subunit volumes based on sex, ALSFRS‐R, and disease duration in ALS patients

After correcting for TIV, hypothalamic subunits were analyzed based on sex, the ALSFRS‐R score, and disease duration in patients with ALS. There were no significant differences in the volumes of several hypothalamic areas between males (16/24) and females (8/24). Furthermore, there was no significant difference in the volume of the hypothalamic subunits between patients with ALSFRS‐R scores ≤41 and >41, disease duration ≤9 months or less, and disease duration greater than 9 months. The ALSFRS‐R score, disease duration, age, sex, and BMI were matched between the two groups for all comparisons.

## 4 DISCUSSION

The study confirms volumetric alteration in the hypothalamic subunit in patients with ALS compared with healthy controls, revealing atrophy of the left a‐sHyp hypothalamic subunit, which is supported by previous research. Furthermore, our findings contribute to the increasing body of evidence that ALS is not exclusively a motor neuron disease (MND), but also encompasses non‐motor/extra‐motor symptoms.^2^, ^29^, ^30^, ^31^, ^32^, ^33^, ^34^ The absence of significant differences in demographic parameters such as age, sex, and BMI between patients with ALS and HCs in our sample provided a level of homogeneity, allowing for a more precise interpretation of the differences in hypothalamic volumes attributable to ALS pathology rather than confounding demographic variables.

### 4.1 General scheme

ALS, traditionally a neurodegenerative disease affecting motor neurons, also includes non‐motor symptoms such as significant changes in metabolic state, eating behavior, altered energy metabolism, and cognitive processes.^3^, ^30^, ^33^, ^35^, ^36^, ^37^ This state impacts patient well‐being and survival, and one of the investigated causes includes altered hypothalamic physiology.^15^, ^17^, ^23^, ^38^, ^39^ Notably, our findings on the atrophy of the left a‐sHyp in the presumably dominant hemisphere in ALS patients replicated and supported previous findings,^22^, ^23^ marked overall hypothalamic atrophy and disrupted connectivity,^7^, ^15^, ^17^, ^21^, ^39^, ^40^, ^41^ and bvFTD,^42^, ^43^ indicating the specific vulnerability of this region. This finding specifies a‐sHyp involvement and highlights a target for further studies. Interestingly, our study found significant correlations between right a‐sHyp volume and disease duration. Our study identified a positive correlation between the volume of the right a‐sHyp and disease duration in patients with ALS, which was unexpected compared with the usual negative correlation between volume and disease progression. Several factors could explain this finding. In the early stages, ALS may affect cellular structures without causing significant volume loss. For instance, increased oxidative stress levels can harm the functioning of mitochondria, worsen endoplasmic reticulum stress, and affect mechanisms responsible for maintaining protein homeostasis. This, in turn, can lead to cell damage and neuronal loss.^44^, ^45^ Moreover, the brain might be attempting to compensate for neurodegeneration by enlarging a‐sHyp, but this effort may be insufficient. Further research is needed to fully understand the mechanisms underlying this observation and its potential implications for understanding ALS pathophysiology.

### 4.2 The involvement of hypothalamic nuclei

Oxytocin (OXT) is a neuropeptide that plays an important role in the regulation of social cognition and emotional behaviors such as anxiety and depression.^46^ This hormone is produced by magnocellular neurosecretory cells located in the supraoptic and PVN (which house oxytocin‐producing neurons).^47^ a‐sHyp, which encompasses the PVN, exhibits notable volume loss in ALS,^22^, ^23^ as shown in our study. These findings support the presence of distinct neuropeptide expression abnormalities that could potentially contribute to the pathogenesis of specific cognitive and behavioral symptoms within the spectrum of this disease.^22^, ^48^ Furthermore, our research has indicated that the OXT pathway may be affected in patients with ALS due to atrophy of the left a‐sHyp subunit, leading to psychiatric symptoms, such as cognitive impairment.^48^ However, additional cognitive and behavioral assessments, along with imaging studies, are required to validate these findings.

Emotional well‐being in patients with ALS slows disease progression, but does not improve survival; therefore, addressing stress is crucial for holistic care and improved outcomes.^49^ Stress is regulated by various nuclei in the brain, including the PVN, dorsomedial nucleus (DMH), and periventricular zone.^50^ The PVN controls stress responses and the autonomic and neuroendocrine components.^51^, ^52^ The PVN, the principal integrator of stress signals, modulates various functions, such as cerebral blood flow, food intake, glucose metabolism, and cardiovascular, renal, gastrointestinal, and respiratory functions.^50^, ^51^ In addition, the amygdala, along with its connections to the hypothalamus, plays a crucial role in emotional processing and influences autonomic, neuroendocrine, and motor systems.^6^ Moreover, the hypothalamus forms connections through the fornix, stria terminalis, ventral amygdalofugal bundle, dorsal longitudinal fasciculus, medial forebrain bundle, and mammillotegmental and mammillothalamic tracts.^6^ This finding also indicates that reciprocal atrophy in the connected regions may induce atrophy in the PVN and the preoptic area. These connections suggest a possible shared vulnerability or disease mechanism, given the involvement of these regions in emotional processing and memory formation alongside hypothalamic functions.

PostHyp, which includes the mammillary body, lateral hypothalamus, and TMN,^26^ has been rediscovered as a crucial hub for behavioral and cognitive processes such as reward‐seeking, exploration, and social memory.^53^ Notably, the supramammillary nucleus (SuM) plays a significant role in hippocampal plasticity and adult neurogenesis.^53^ Furthermore, the hippocampus‐PVN connections are pivotal for stress response, memory, emotional regulation, and dysfunction in these connections.^51^, ^54^, ^55^ Furthermore, SON and PVN nuclei, located in the bilateral anterior hypothalamus and SCN, undergo structural and functional changes during aging.^56^ These nuclei, along with their connections, are affected in various ways by neurodegenerative diseases. SON and PVN, the hypothalamic–pituitary axes, and peptide hormones are produced in these nuclei in healthy aging and a range of neurodegenerative diseases, including ALS and FTD.^56^, ^57^ As individuals age, these nuclei experience sex‐based differences, such as changes in size and vasopressin and corticotropin‐releasing hormone levels, likely due to hormonal changes. In contrast, oxytocinergic cells and thyrotropin‐releasing hormone levels remain stable.^56^ These findings highlight the importance of our research, which shows a relatively strong correlation between the volume reduction in the right supTub (location of the dorsomedial nucleus, PVN, and lateral hypothalamus) and left postHyp (encompassing the mammillary body and TMN) with age for future studies. The findings also suggest that the PVN and mammillary body likely communicate within the broader hypothalamic circuitry, contributing to memory and spatial processing, as well as playing regulatory roles in physiological functions such as the dopaminergic system. However, with continued investigation, future research has the potential to expand our knowledge of the functions of the PVN and mammillary bodies, and their connections.

## 5 LIMITATIONS AND RECOMMENDATIONS

This study had certain limitations that are worth noting. Our study had a relatively small sample size. It is impossible to define functional subnuclei within the hypothalamus,^26^ which may have affected the accuracy of our results. Additionally, we could not use neuropsychological tests with imaging because of the lack of a comprehensive literature on this topic. These limitations may have implications for the interpretation of our findings, and should be considered when evaluating the conclusions of this study.

Future research may explore structural and functional connections between the subregions of these structures and hypothalamic subregions using other automated methods based on FreeSurfer for volume segmentation, enabling a better understanding of the findings and potential changes.

The hypothalamic system receives and integrates inputs from the brain to maintain a balance among feeding, energy storage, and expenditure. Disruption of these inputs may drive metabolic imbalances. The architecture of large‐scale networks is disturbed in ALS, and ALS‐related pathobiology can affect a significant proportion of cortical and subcortical structures that evolve with disease progression.^7^, ^21^ We recommend that future studies use other neuroimaging measures, including the integration of structural and functional connectivity, to elucidate the probable disruption of projections to distinct parts of the hypothalamus.

Exploring the issue of volume loss in the hypothalamic subunit should be considered in patients with ALS to reduce the impact of the disease. As ALS deteriorates, particularly in the growing elderly population, it is important to address potential comorbidities.^58^ It seems crucial for future studies involving multiple centers should investigate the connection between ALS and the endocrine system in larger groups of patients. Furthermore, future research should focus on the neuropsychological situation experienced by patients with ALS and its impact on the hypothalamic nuclei.

## 6 CONCLUSIONS

This study contributes to the understanding of ALS‐related changes in the hypothalamic subregion and provides evidence for the involvement of hypothalamic regions in the pathophysiology of ALS. Our study revealed a significant reduction in the volume of the volume of the left a‐sHyp subunit. This supports previous research and highlights that ALS is a disorder that affects multiple regions of the brain beyond the motor regions. These findings open avenues for further research on the clinical implications of hypothalamic atrophy in ALS and its potential use in the development of targeted therapeutic interventions.

## CONFLICT OF INTEREST STATEMENT

The authors declare that they have no conflicts of interest.

## Data Availability

The data supporting the findings of this study are available from the corresponding author [SB] upon reasonable request.
